# *Eichhornia crassipes* (Mart.) Solms: Uses, Challenges, Threats, and Prospects

**DOI:** 10.1155/2020/3452172

**Published:** 2020-07-07

**Authors:** Opeyemi I. Ayanda, Tolulope Ajayi, Femi P. Asuwaju

**Affiliations:** ^1^Department of Biological Sciences, Covenant University, Ota, Ogun State, Nigeria; ^2^Department of Aquaculture and Biotechnology, National Institute for Freshwater Fisheries Research, New Bussa, Niger State, Nigeria

## Abstract

Water hyacinths pose serious challenges to humanity and the environment. Considering the enormity of the menace associated with the growth and spread of the plant and the difficulty in achieving a single, generally acceptable control method, it is becoming increasingly imperative to explore the potentials of the plant. New water hyacinth-related articles are regularly being published. Recently published articles about the plant were accessed, and the information in these articles is presented in the context of the pros and cons of the plant. Some of the benefits that can be derived from the plant include biogas and biofuel production, medicinal functions, vermicomposting, compost production, and bioremediation. However, clogging of waterways, obstruction of water transportation, and fishing activities; breeding grounds for pests and diseases; and reduction of water quality, loss of biodiversity, and economic downturn in areas invaded by the plant are problems associated with it. The peculiarity in the invasiveness of each situation should determine whether or not the growth of the plant is a problem, especially if the opportunity to harness the potentials of the plant exists. There are three major methods for controlling the plants when control becomes inevitable: mechanical, chemical, and biological. To achieve the best control, integrating two or more control methods is advised.

## 1. Introduction

Certain aquatic weeds can negatively impact the environment as well as the livelihood of humans. Among common aquatic weeds, *Eichhornia crassipes* (water hyacinth) remains the most widely distributed and vicious aquatic weed [1]. Water hyacinth has become the world's most invasive weed due to its rapid spread, ecological adaptability, and negative impacts it causes on the environment, economic development, and human health [2]. Water hyacinth derives its origin from the Amazonia basin in South America and holds the reputation of being one of the most strenuous water weeds to manage [3]. It has spread from the Amazon basin to many tropical and subtropical regions of the world [4, 5]. It has been reported in Brazil, Argentina, Paraguay, Venezuela, and Chile [6]; El Salvador, Panama, Costa Rica, Mexico, Portugal, Spain, Israel, Italy, Japan, India, and Indonesia [7]; the United States of America and Virgin Islands [8]; and several parts of Africa including Nigeria, Ethiopia, Kenya, South Africa, Zambia, and Zimbabwe. The ability of the plant to grow in different aquatic ecosystem types including lakes, streams, ponds, waterways, and ditches and in cold and warm climates enables the plant to challenge the ecological stability of these water bodies [9]. It has been a menace not only to aquatic biota but also to the communities where it is found. By obstructing sunlight, water hyacinth affects the productivity of phytoplanktons and other macrophytes, indirectly affecting the productivity of other organisms that dwell in the aquatic ecosystem and ultimately leading to a reduction of the biodiversity of the environment which it occupies [10]. Unlike other submerged flora and phytoplankton, the plant does not discharge oxygen into the aquatic ecosystem. This results in a decrease in the dissolved oxygen concentration of the water body.

Over the years, different authors have reviewed water hyacinth to varying degrees. Edwards and Musil [11] discussed the biology, ecology, and some benefits of the plant. Considering how long ago this review has been done, many more beneficial uses of the plant have since been reported. Fessehaie [12] reviewed the status of the weed vis-à-vis its negative impacts on the aquatic ecosystems in Ethiopia. Villamagna and Murphy [13] comprehensively reviewed mainly the effects of water hyacinth on the aquatic ecosystem as a whole. Coetzee et al. [14] reviewed the biological control programs being undertaken against water hyacinth in South Africa. Priya and Selvan [15] also discussed the remedial abilities of the weed and suggested how the weed can be modified/improved upon for better remediation. This review discusses the combined benefits and negative aspects of water hyacinth, using recently (mostly within the last five years) published articles with comprehensive and up-to-date information. It further discusses the future potentials or threats (as the case may be) of the weed.

### 1.1. Morphology and Characteristics of Water Hyacinth

There are about seven species of water hyacinth. It is a widely established attractive aquatic plant having circular to oval-shaped leaves with malleable covered petioles and carrying aesthetic lilac to blue in color flowers [16]. The fully grown plant comprises long, pendant roots, rhizomes, stolons, leaves, inflorescences, and fruit. The presence of air-filled sacs in the leaves and stems enables them to float on the surface of the water [17]. The plant can grow to a height of up to 1 m, with an average height of about 40–60 cm. The plant's inflorescence can support 6–10 flowers similar to the lily, with each flower being 4–7 cm in diameter [1]. The plant's ability to float also enables it to grow in harsh conditions such as moist sediments for long periods [18].

Under suitable climatic conditions such as temperature and humidity, *E. crassipes* blooms throughout the year and it possesses the capability of increasing its population between 12 and 14 days in Nigeria [19]. Studies by Akinyemiju and Bewaji [20] suggested that the outbreak of water hyacinth in rivers and lakes reaches its climax between the months of June and September with the highest prevalence being in July. Reproduction in the plant may be by vegetative means, occurring through the generation of stolons. Sexual reproduction can also be initiated through seeds, which are capable of existing in water for six years, thus making water hyacinth tough to eradicate. The population of water hyacinth can be doubled in a week, in a favorable environment [21]. The prevalence of water hyacinth in aquatic environments that are nutrient-rich can create thick grass mats surrounding a vast area of water. The large, dense, lustrous, and ovoid leaves of the plant can grow above the top of the water body as high as 1 m. The plant possesses lengthy, bulbous, and spongy stalks [22]. The loosely hanging fibrous and feathery roots are purple and black. An upright stem bears 8–15 distinct attractive pink to lavender flowers, each with six petals. The most widespread among the seven species of water hyacinth is *Eichhornia crassipes*. The plant can be divided into three major portions: a brown fibrous root mostly below the water surface that creates the base structure of the weed, a green partly tender stalk, and plump green photosynthetic leaves [22]. The ecological, morphological, developmental, and biological attributes of water hyacinth include its ability to adapt to a wide range of ecological conditions, its growth being easily stimulated in the presence of nutrients especially with excess nitrate and phosphate concentrations, its high rate of vegetative growth and reproduction, its production of seeds that can remain viable for very long periods (up to 15 years) and of 4 daughter plants (from each mother plant) which possess the ability to reproduce after two weeks, and the absence of any known natural adversary of its seeds and its ability to develop into mats of up to 2 m thick [17].

### 1.2. Taxonomy

Water hyacinth, an indigenous monocot aquatic plant of Brazil and Ecuador region of South America, is associated with the group of plants called lilies [23]. It is commonly known as the floating/common water hyacinth. It belongs to the genus *Eichhornia*, which comprises seven species: *E. natans*, *E. heterosperma*, *E. crassipes*, *E. azurea*, *E. diversifolia*, *E. paniculata*, and *E. paradoxa*. Among the seven species, only two species are prolific in Africa, namely, *E. natans* and *E. crassipes*, the latter being abundant in Nigeria [1]. *Eichhornia crassipes* is distinguishable from other similar aquatic plants by its lustrous leaves, the partial lump of its petioles, and its distinct purple flowers. Water hyacinth is classified as follows:

  Domain: Eukaryota
  Kingdom: Plantae
  Phylum: Spermatophyta
  Subphylum: Angiospermae
  Class: Monocotyledonae
  Order: Pontederiales
  Family: Pontederiaceae
  Genus: Eichhornia
  Species: *Eichhornia crassipes* [24]

### 1.3. Water Hyacinth Biomass

A variety of compounds are present in a freshwater hyacinth plant. The biomass of water hyacinth in Nigeria's aquatic environment has been estimated at between 28.8 and 32.2 tons of dry matter/hectare/year [25]. The ethanolic and methanolic extract of water hyacinth revealed the presence of the following compounds: alkaloids, flavonoids, phenols, carbohydrates, proteins, amino acids, tannins, terpenoids, saponins, and glycosides [26–28]. Furthermore, an analysis of the dried biomass of water hyacinth by Sivasankari and Ravindran [29] is as reported in Table 1.

Ogamba et al. [30] also reported average proximate values of 84.5%, 6.5%, 4.3%, 2.4%, 6.4%, and 14.25% for moisture content, ash, protein, lipid, fiber, and dry matter, respectively, in water hyacinth.  Table 2 shows the composition of metabolites in water hyacinth as reported by Tulika et al. [31].

Water hyacinth comprises the following elements: C, O, Na, Mg, Al, Zn, K, Ca, Fe, P, and S, with carbon and oxygen having the highest percentages, making it a good source of biofuel for feedstock [32, 33]. Other major elements were calcium (Ca), potassium (K), and silicon (Si) [28]. In the leaves, potassium and calcium were the most common elements present, while potassium, calcium, and chlorine were present in the stems in increased quantities. The roots of water hyacinth act as phytoaccumulators, whereby the roots absorb toxic pollutants and chemicals at higher concentrations in water bodies [34].

### 1.4. Economic Importance of Water Hyacinth

*Eichhornia crassipes* is an invasive weed found in several aquatic ecosystems all over the world: lotic, lentic waters, creeks, and creeklets [30]. The intense rate of proliferation of *E. crassipes* results in a change in the chemistry of water and disruption of aquatic biota, thereby rapidly increasing water loss due to evapotranspiration. The threat the weed has posed to biodiversity has been described as alarming and lately, attention is being given to using it for numerous other purposes [35]. The weed can impact the health of aquatic ecosystems and the safety of the water environment thereby causing significant potential ecological and environmental risks [21]. Nevertheless, the plant can be exploited for a variety of beneficial uses. The plants' biomass is useful for bioremediation and bioaccumulation of hazardous pollutants and metals; for production of biogas and biofuel; as a source of feed for fishes and animals; as a carbon source for microbial metabolism, in vermicomposting and compost production; and in medical and several other essential aspects [2]. The economic importance (positive and negative) of water hyacinth across several regions is discussed hereinafter.

### 1.5. Bioremediation (Phytoremediation)

Phytoremediation can be defined as a set of methods that uses plants for cleaning up soil or water so that either is free from both organic and inorganic impurities [36]. *Eichhornia crassipes* has been found to be a suitable candidate for bioremediation, implying that there are certain characteristics the plant possesses that favors its use in this technology. Several researches have indicated that water hyacinth, under controlled growth conditions, is an efficient and economical substitute to conventional cleanup methods as it speeds up the extraction and absorption of industrial and agricultural effluents contaminated with organic, inorganic, and toxic metals. Water hyacinth is capable of treating wastewater containing heavy metals and dissolved ions [37]. The leaves, roots, and bulb tissues of the plant exhibited hyperaccumulation abilities for heavy metals [38]. Anudechakul et al. [39] reported that the interaction of *E. crassipes* and *Acinetobacter* sp. strain WHA is a very promising economically viable alternative to removing and degrading chlorpyrifos, a pesticide from polluted water. Water hyacinth has shown the ability to reduce the concentration of heavy metals, dyestuffs, and even water physicochemical parameters [15]. *E. crassipes* has also been found positive to bioremediate soil contaminated with crude oil and Total Petroleum Hydrocarbons (THC) [40]. In Malaysia, it has shown potential for removing palm oil from palm oil mill effluent (POME), with a shorter time of treatment and an ability to reduce biological oxygen demand from the effluent to meet regulatory standards [41]. Water hyacinth has been deployed as a bioindicator of heavy metal pollution in a river and wetland in Pakistan [42]. The different parts of the weed were reported to have a high bioconcentration factor index for the pollutants analyzed. This indicates that water hyacinth is a very good plant for rhizofiltration process, a technology that is being explored as a means of controlling pollution. It has been reported from the East Kolkata Wetland in India to effectively take up heavy metals like Cu, Zn, and Pb in its body tissues [43]. The heavy metal content, carbon, and biological oxygen demand for toxic wastewater decreased many folds after remediating with water hyacinth. This demonstrates the potential of water hyacinth to reduce the toxicity of wastewater via phytopurification. [44]. In the Sukinda chromite mines (SCM) in India, the removal of toxic hexavalent chromium from wastewater was experimented using water hyacinth. Up to 99.5% of chromium removal was achieved with about 100 L of wastewater in 15 days. In the same study, water parameters like total dissolved solids and biochemical oxygen demand were greatly reduced in the toxic water [45]. Saha et al. [46] further reported that water hyacinth was also able to remediate cyanide from blast furnace water by as much as 95% in three days. Wenwei et al. [47] reported the ability of water hyacinth to actively remove nitrate in agricultural eutrophic wastewater. The plant has the capacity to store excess nitrate in its root and shoot system.

### 1.6. Vermicomposting

The process of using earthworms to transform organic biomass into nutrient-rich compost that mainly comprises earthworms with a mixture of decomposed organic matter is called vermicomposting. The resulting compost functions as a eutrophic natural manure and soil stimulant [48]. Vermicompost is obtained when organic waste decomposed by microbes in the digestive tracts of earthworms [49]. Water hyacinth has been found to be a very good vermicomposting raw material. In combination with cow dung, water hyacinth was successfully converted into vermicompost in Zimbabwe [50] but, in fact, was found to yield better vermicompost when used alone as a substrate for earthworms [49]. Vermicomposting *E. crassipes* from Lake Victoria in Kenya yielded richer vermicompost [51]. Vermicomposting using water hyacinth mixed with cow dung significantly improved the growth of *Arachis hypogaea* planted in the field [52]. Water hyacinth vermicomposted with *Lactobacillus sporogenes* led to an increase in nutrient composition of the vermicompost after 60 days, suggesting that the weed could be a good biofertilizer [53].

### 1.7. Compost Production

Composting remains among the most efficient methods of producing biofertilizers from water hyacinth. The presence of inorganic compounds such as nitrogen (N) and phosphorus (P) in the roots of the plant makes it an appropriate raw material for inorganic fertilizers and compost manufacture. Composting can also help to largely reduce the application of chemical fertilizers on agricultural fields, thus providing an eco-friendly agricultural approach. The quality of compost from a mixture of over 10,000 kg of water hyacinth and poultry litter is reported to be above quality standards [54]. Compost made from water hyacinth alone has been shown to make better quality compost than compost made from a combination of water hyacinth and manure which suggests that composted water hyacinth could be used as an alternative to peat in substrates for nurseries [55]. Using water hyacinth compost, therefore, could be a way of preventing secondary pollution. Compost made of water hyacinth, cow dung, and sawdust in the ratio 6 : 3 : 1 in India led to an increase in all nutrients (N, P, Na, K, and Ca) tested and improved compost stability, indicating that water hyacinth is a good raw material for compost production [56]. Organic compost produced from water hyacinth in the Lake Victoria basin in Kenya has been suggested as a very suitable option for controlling acidic soils while simultaneously replenishing soil elements [57]. Mixtures of water hyacinth manufactured into agricultural fertilizers from several forms of animal wastes, plants' wastes, house-hold and domestic wastes, etc. are utilized to improve water retention in soils in land-locked areas [58].

### 1.8. Fish Feed

Fishes have different feeding habits and some species such as tilapia and grass carp can feed on several aquatic plants. Hence, water hyacinth can be used as a raw material in formulating fish feed. This may increase the crude protein level of the feed (due to the high protein content in the leaves and roots of the plant) and possibly in digesting the feed better. Diet consisting of up to 40% of water hyacinth may be used to replace fish meal in diet formulation for common carp fry. Fries of common carp fed with fish diet inclusive of water hyacinth were reported to show a significant weight increase after 70 days [59]. Sarker and Aziz [60] observed alterations in the supply of nutrients in fishes by the addition of water hyacinth to fish feed. It reduced the cost of feed formulation, thereby raising profit margins. Results indicated that the addition of up to 25% water hyacinth in the composed feed did not show any negative impacts on the development of mirror carp but that incorporating about 15% water hyacinth meal in a diet was best for mirror carp. In evaluating the potentials of water hyacinth as an alternative feed for grass carp *Ctenopharyngodon idella* using whole plant and plant parts, Mahmood et al. [61] reported a higher fish weight gain, higher crude protein, and better nutrient digestibility in the leaf meal diet without any histological alterations in fish liver and kidney. This indicates that the leaf of water hyacinth is a very suitable option as an alternative aqua feed. Water hyacinth has also proven to be a food strategy in angelfish diets and may be implemented in the fish's diet at up to 32% levels of crude protein. Improved growth performance and survival of angelfish, *Pterophyllum scalare,* was reported with the inclusion of water hyacinth biomass in the diet of the fish [62].

### 1.9. Biofuel Production

Increasing industrialization and urbanization is rapidly exhausting earth's nonrenewable energy resources necessitating the need for new ways of producing fuel that can be readily available, affordable, and environmentally friendly. Biofuels are any energy-enriched chemicals that are produced via biological means or derived when the biomass of once-living organisms is chemically converted [63]. The low lignin content present in water hyacinth makes the plant a great choice when sourcing for biomass, as cellulose and hemicellulose are converted with ease to sugar that is fermentable, resulting in great quantities of biomass that can be exploited in the biofuel industry. Biodiesel production using potassium hydroxide extracts from water hyacinth has been reported [64]. Bhattacharya et al. [65] did an anatomical study of water hyacinth and adopted methodologies for the production of xylose from it which can then be converted to xylitol, poly-alcohol obtained from hemicelluloses. It was reported to be a very promising biological process, being economically viable and eco-friendly. Pretreatment of water hyacinth with dilute sulfuric acid (10% w/v and 2% v/v, respectively) at high temperature and pressure was simultaneously studied with the economic assessment of the process for enzymatic saccharification [66]. The study reveals an economic prospect for water hyacinths as a means of generating biofuel.

The use of water hyacinth as an alternative source of bioenergy production was further demonstrated by Ruan et al. [67]. The authors analyzed water hyacinth biomass using the Van Soest method and developed an effective alkali pretreatment method for water hyacinth enzymatic hydrolysis to yield reducing sugars. It was reported that, with favorable hydrolysis conditions for the alkali-pretreated water hyacinth biomass, the rate of cellulose conversion was up to 100%. In India, bioethanol production achieved through SSF of NaOH/H_2_O_2_-pretreated water hyacinth has been reported to be a promising strategy for biofuel production [68]. It was reported that maximum ethanol concentration was achieved with saccharification and fermentation (SSF) from pretreated water hyacinth at 35°C two times more than that produced by *Saccharomyces cerevisiae* at 30°C. Very recently, an ex-ante cost-benefit analysis of the production of bioethanol from water hyacinth was conducted by Wang et al. [69]. They reported that compared with the active control approach of mechanically collecting water hyacinth and using it for landfilling, there is the economic feasibility of using the collected biomass for the production of bioethanol. Reports of studies attempting to use water hyacinth for manufacturing biodiesel from also exist in literature [70].

### 1.10. Animal Feed Production

The dry matter of water hyacinth contains high protein and mineral content, making it a useful source of animal feed and roughage [21]. The presence of some important phytochemicals such as alkaloids and saponins has been reported in water hyacinth, suggesting that they can be considered as good feed supplement [71]. The nutritive value of the combination of water hyacinth and guinea grass using different treatment combinations as a source of animal feed was reported by Mako et al. [72]. Inclusion of 30% water hyacinth in animal feed was reported to give optimum performance and the weed was suggested to be a good forage material. A pilot study on the potential of water hyacinth as an alternative to conventional components of animal feed shows that it increases production by 30% and reduces cost by 20%. Furthermore, incorporating water hyacinth into animal feed was reported to increase milk production in cattle by up to 20%. In an experiment conducted to observe the growth performance of pigs fed with feed incorporated with water hyacinth, a lower feed cost/body weight gain was reported. Furthermore, the protein content of the weed has a high digestibility [73]. Indulekha et al. [74] conducted a study aimed at converting water hyacinth into silage to be used as animal feed using wilted and freshwater hyacinth in combination with molasses. It was reported that wilted hyacinth with up to 5% molasses or 10% cassava yielded the best result in terms of pH, odor, and palatability of the feed. Wimalarathne and Perera [75] also opined that there is a great opportunity of utilizing water hyacinth to reduce livestock feed shortage especially in areas where there is scarcity. Furthermore, they reported that other control methods notwithstanding, using water hyacinth as a livestock feed, can be a better approach to controlling the invasiveness of the plant.

### 1.11. Biogas Production

Biogas is the product of fermentation of organic materials in the absence of oxygen using microorganisms. Water hyacinth can be useful in the production of biogas because it possesses a high amount of hemicellulose contents [76]. Attention is now being drawn toward the production of biogas as a source of fuel due to its cleanliness and cost. About three liters of biogas was reported [77] to be produced when 2.5 kg of dried biomass of water hyacinth was mixed with cow dung and poultry droppings in the ratio 2 : 2 : 1 when the potential of water hyacinth to produce biogas was studied. Methane and carbon dioxide were produced up to 62% and 34%, respectively. Biogas yield of between 70% and 75%, using water hyacinth, has been reported by Njogu et al. [78]. This yield is said to be high enough to power internal combustion engines coupled with an electricity generator. Another study compared the potential of three different plants—giant reed, water hyacinth, and maize—codigested with poultry waste for biogas generation. Water hyacinth was reported to generate the highest volume of biogas, suggesting that it might be a good substrate for biogas production at the community level [79]. Codigestion of water hyacinth and fish waste in the ratio 1 : 2 has been shown to produce as much as 0.56 liters of biogas with over 70% composition of methane [80]. Rathod et al. [81] also reported biogas production from water hyacinth. Measurement of daily gas production indicated about 58% methane and 42% carbon dioxide gas production. The experiment generated biogas in such quantity that can be used for lighting and cooking implying that water hyacinth is a good raw material for biogas production. Pretreatment of water hyacinth with pure culture isolates of *Citrobacter werkmanii* VKVVG4 was reported to produce biogas three times more than untreated water hyacinth [82]. Chemical pretreatment of water hyacinth with H_2_SO_4_ concentration of 5% v/v and residence time of 60 min was reported to produce biogas by as much as 130% more than without pretreatment [83]. Electrohydrolysis pretreatment of water hyacinth at 20 V for 60 min for 30 days has been reported to reduce hydrolysis time and to also increase biogas production as compared with untreated water hyacinth [84]. Production of biogas from a mixture of water hyacinth and cow dung was reported by Bote et al. [85]. They reported that the gas generated was of high methane content and enough to power a gas generator.

### 1.12. Medicinal Functions

Water hyacinth is reported to have anti-inflammatory, antifungal, and antibacterial functions [86]. Furthermore, it can be used as a hair fragrance, to treat cholera, sore throat, and snake bites. Pandey et al. [87] reported that the roots, leaves, and flowers of water hyacinth have been scientifically tested to contain chemical constituents that are able to cure ailments. Water hyacinth extracts possess the potentials to be used as an alternative for antiaging as demonstrated by Lalitha and Jayanthi [88]. Water hyacinth was reported to demonstrate anticancer ability due to the presence of some cancer-fighting compounds [89]. Two dermal creams formulated with ethyl acetate extracts of water hyacinth were analyzed for antiaging properties through DNA damage inhibition and DPPH radical scavenging assays. It was noted that there was an increase in the DNA inhibition and DPPH radical scavenging properties with increasing concentration of both creams, inspiring confidence about the utilization of water hyacinth in the cosmetics and beauty industry [88]. Furthermore, Guna et al. [90] reported the ability of parts of water hyacinth, in powdery form, to control the release of the drug metformin, suggesting it to be a potential rate-retarding material. The larvicidal, antitumor, and wound healing properties of water hyacinth have also been reported [91]. Lenora et al. [92] also reported that *in vitro* studies on human cervical cancer cell lines (HeLa) using methanolic extracts of water hyacinth showed anticancer potential. It was observed that MTT assay analysis showed inhibition of cell growth. Haggag et al. [26] also reported the fungicidal and bactericidal actions of the extracts of water hyacinth. The leaf extracts of water hyacinth have been established to possess phytochemicals with potent antioxidant and hepatoprotective activities in vitro [93]. The ability of water hyacinth extracts to reduce blood lipid levels, thereby preventing fatty liver, has been reported [94]. The water hyacinth extracts can be made into a clinically acceptable solvent and used for the treatment of lipid disorder or for the treatment of patients with fatty liver. The water hyacinth extracts have also be found useful as health-care products.

### 1.13. Potential Rural Socioeconomic Benefits

Ropes and yarn can be made from the stem of water hyacinth [95]. This can be processed further into baskets, hats, mats, and even furniture. Production of paper and cardboard from the weed can also be considered. For better quality, water hyacinth fiber may be blended with up to 50% jute or waste paper. Inhabitants of communities occurring around water bodies where this plant grows can begin to process the plant into useful products for income. Additionally, fiberboards can be made from it by small-scale industries and this can be used for indoor partitioning of ceilings and walls.

### 1.14. Effects on Water Transport, Irrigation, and Hydropower

Water hyacinth disrupts water transportation. The weed forms thick mats that cover the surface of water bodies, preventing people's access to schools, communication, health facilities, fishing grounds, and even local markets. Mbula [96] reported that, in the Kafubu river in Zambia, precious time is wasted in setting the fishing gears in water due to the presence of the plant's mats, while the weed also reduces the amount of water needed for irrigation and blocks channels of irrigation. The proliferation of water hyacinth is reported to significantly hamper fishing and fish trading activities in the neighborhood of River Tano in Ghana [97]. The effects of the invasion on fishing and fish trading included a reduction in fish quality and catch, difficulty in using fishing gears, reduced profit, and increased cost of fishing. Arp et al. [98] valued losses from agricultural water, useful for irrigation systems to be in the region of about R54 million per annum. This water loss is due to evapotranspiration from the invasive weed, water hyacinth. This highlights the humongous and detrimental effects the invasiveness of water hyacinth can have in economically productive water resources, which by extension can negatively impact a nation's agricultural sector. Worku and Sahile [99] reported the impacts of water hyacinth on Lake Tana in Ethiopia. The lake is said to provide economic benefits to individuals living in its surroundings in the form of fishing and recreational facilities. Invasion of this lake by water hyacinth is said to be gradually taking away this source of livelihood from residents around the lake by hampering transportation on the lake. Honlah et al. [100] conducted a cross-sectional research on the impact of water hyacinth on the movement of students who use River Tano and Abby-Tano Lagoon in Ghana as a means of transportation. The invasion of the water bodies by water hyacinth invasion was reported to impede the smooth transport of the students so much so that they may have to remain at home during the peak of the weed invasion, thereby negatively affecting the human resource base of the people. Hydropower systems are rendered inefficient from the impacts of water hyacinth. Owen Falls hydropower system on Lake Victoria has been affected by the invasion of this aquatic weed, and power generation is reduced due to the weed's fast growth and multiplication [101].

### 1.15. Reduction of Water Quality and Biodiversity

The highly reproductive nature of water hyacinth poses a great threat to biodiversity as the weed easily outcompetes other species. Mengistu et al. [102] compared the impact of water hyacinth on the assemblage of macrophyte species in infested and noninfested parts of Lake Abaya, Ethiopia. It was reported that water hyacinth affects the composition of macrophytes, their abundance, and diversity so much so that in extreme instances, the community of the macrophytes shrank into single flora. In Nigeria, Chukwuka and Uka [103] reported that species of rotifers, cladocerans, and copepods were significantly reduced in abundance due to the infestation of Awba Reservoir by water hyacinth. Water hyacinth is able to grow to about 1.5 meters above the water level with the necessary nutritional requirements and it can double this size in just two weeks [104]. Water hyacinth, when it grows explosively, forms mats over the water surface. This reduces the amount of light reaching other submerged plants, while using up much of the dissolved oxygen content in water in the process. The reduced dissolved oxygen content will further impact the diversity of plankton and other aquatic biotas. This will lead to a shift of species, from those demanding high oxygen to those that can tolerate low oxygen [104]. This could also lead to the death of those species that cannot adapt under low oxygen conditions. Furthermore, there is an overall reduction in productivity, due to the inability to access light for photosynthesis. This consequently reduces biodiversity. The invasiveness of water hyacinth can also reduce water quality either for domestic purposes by humans or for the wellbeing of other aquatic biotas. The turbidity of water increases in water hyacinth-infested waters, and this can consequently increase temperature and affect other water parameters. Tobias et al. [105] studied the impact of water hyacinth treatment on tidal systems. It was reported that water quality parameters such as dissolved oxygen, temperature, and turbidity were negatively impacted. Additionally, reduced concentrations of dissolved oxygen catalyze the discharge of phosphorus from the deposits present at the bottom of the water body which as a result speed up the rate of eutrophication, favoring and enhancing algal blooms [106].

### 1.16. Breeding Ground for Pests and Vectors

The abundance of water hyacinth on River Tano and Abby-Tano Lagoon in Ghana was reported to be a habitat for vectors of disease-carrying organisms such as mosquitoes, thereby increasing the incidence of malaria infection in the communities around the two water bodies [100]. Water hyacinth has also been implicated in the transmission of schistosomiasis. Vectors of schistosomiasis snail species of the genus *Biomphalaria* and *Bulinus* have been reported to use the leaves of the weed as resting sites. *Bulinus africanus* and *Biomphalaria sudanica* were found to attach preferably to water hyacinth in Lake Victoria than to their native hippo grass [107]. Feikin et al. [108] reported that there exists a very strong association between the number of cholera cases reported in the Nyanza Province of Kenya and the yearly water hyacinth coverage of Lake Victoria. This, in essence, suggests that water hyacinth might contribute to the outbreak of cholera thereby causing sporadic disease in the East African region. A marked difference was noticed in the *Escherichia coli* count in areas infested with water hyacinth and areas that are not around Lake Victoria [109] suggesting that the bacteria find the water hyacinth mats very suitable as a habitat. As the weed has the ability to easily spread, the disease associated with *Escherichia coli* may increase among the riparian communities.

### 1.17. Future Threats and Risks

At the moment, water hyacinth is not a major problem in every part of the world. However, the geographical range of the weed is very wide. In regions where the weed is not invasive presently, the potential exists to the extent that the reverse may occur in many decades to come. This is as a result of the projected changes in climate in the future. According to Kriticos and Brunel [110], the poleward extent of the weed is bounded by cold stress, and its ability to inhabit some tropical areas in Africa is limited by heat stress. If, at any point in the future, these limits are overcome, the invasiveness of the plant will spread further.

### 1.18. Management of Water Hyacinth

Water hyacinth management is of utmost importance. If the weed is not properly managed, aquatic life could suffer. There may not be a single, generally acceptable way of controlling the weed as the peculiarity of its invasiveness in different environments may be different. Getting rid of the weed completely may be difficult to achieve due to its ability to grow fast and spread within a short period of time; however, the sole objective of any control measure should be to eliminate the plant faster than it grows. Awareness and community mobilization on the need to ensure that the growth of the weed does not become a menace should be engaged in regularly. Nongovernmental Organizations (NGOs) on issues relating to the environment should also actively participate in the campaign to effectively manage the growth of the weed. Government agencies and ministries should also take the lead in battling the scourge of the invasiveness of the weed. According to Degega [9], the best control strategy is by preventing its growth in water bodies. The long-term effects of individual control techniques, with continued utilization, should help mitigate the negative impacts of this plant [111]. To achieve more efficiency, a combination of control strategies may be employed. There are three major ways by which water hyacinth is being controlled: mechanical/manual, chemical, and biological control.

### 1.19. Mechanical/Manual Removal

This involves the use of machines and other designated pieces of equipment for removal of the weed from water. Mechanical mowers, destroyer boats, mechanized dredgers, weed harvester tractors, and crusher boats are some of the machines used. The removed weed is moved onto the bed of the equipment. However, it is important to ensure that nontarget species are not impacted by the process of removing the weeds. Mechanical removal can be a prelude to chemical control whereby the water is then sprayed with herbicides after the weeds have been removed. This control method may be expensive considering the nature of the equipment in use. Manual removal does not require any technical expertise, especially when plants are removed from the water body with the use of hands but it is only effective for a small body of water and, hence, inefficient in massive lakes or water bodies. It also has the drawback of being time-consuming. This control method can bring about changes in dissolved oxygen content of water and trophic structure, thus speeding up the rate of eutrophication [112].

### 1.20. Chemical Control Technique

This technique involves the application of herbicides to get rid of the weed. Paraquat and Glyphosate are two chemicals widely used for this purpose especially in Africa [17]. This method of control is quick, efficient, and not costly when compared to mechanical and manual control techniques but it requires a select skill to be efficient [113]. Even though stakeholders in issues concerning the environment advise that caution be taken when chemical control is being considered because of the effects it may have on nontarget organisms, these fears are not always actualized. Other nonherbicidal chemicals are now being used for the control of water hyacinth. Agidie et al. [114] reported the application of acetic acid to kill water hyacinth. The acid was said to have as much efficiency as glyphosate suggesting that it can be used as an alternative since it is more eco-friendly. Other acids like citric acid, formic acid, and propionic acid have also been tested for the control of water hyacinth with varying degrees of efficiency [115].

### 1.21. Biological Control Technique

Biological control alternatives include the exploitation of a host as a distinct natural adversary of water hyacinth to decrease the population size of the weed. To date, some of the natural enemies that have been reported include moths (*Bellura* spp., *Xubida* spp., *Niphograpta* spp.), flies (*Thrypticus* spp., *Megamelus scutellaris*), mites (*Orthogalumna terebrantis*), weevils (*Neochetina* spp.), and grasshoppers (*Cornops aquaticum*). Furthermore, fungal species, *Trichothecium* spp., *Aspergillus* spp., *Trichoderma* spp., *Fusarium* spp., and *Rhizoctonia* spp. were introduced into water hyacinth-infested waters in Lake Tana and they were reported to be very promising as a biocontrol agent of the weed [116]. *Megamelus scutellaris* (Hemiptera) has also been tried with some degrees of success. It is suggested that an integrated approach of this insect with mycoherbicide will enhance the biological control of the weed [117]. Biological and chemical controls could also be integrated. Tipping et al. [118] reported that integrating chemical and biological control doubled the efficiency of the application of herbicides alone for the control of water hyacinth. Biological control is an ecologically safe technique, although it is a difficult process and slow to initiate.

## 2. Future Research, Prospects, and Conclusion

As stated earlier, some medicinal uses of water hyacinth have been reported. Further research may be needed in this regard to explore the possibility of drug formulation from this weed. Considering its use as feed for animals, toxicity studies have to be further investigated to ensure that the plant is safe for use whether as food for animals or as treatment of ailments. A major cause of concern is the ability of this plant to invade different kinds of water bodies across different geographical zones. Molecular analysis of the plant growing in different water habitats and different geographical zones may also be important to know the similarities in the genes across the different habitats. This will provide information on better understanding the invasiveness of the plant. This way, the focus can shift to genetically modifying the plant to be less invasive.

Unarguably, the invasive nature of water hyacinth makes it an environmental challenge; however, the prospects of the weed can also not be ignored. It may be better not to generalize the weed as a menace but rather to look at the peculiarity of each situation where the weed grows. In situations where facilities exist to utilize the potentials of this plant, efforts should be geared toward harnessing and optimizing its growth. Where the reverse is the case, a combination of the most effective control method that is not too slow and cost-effective should be considered in managing the growth of the weed.

## Figures and Tables

**Table 1:** Analysis of dried biomass of water hyacinth.

S.no.	Dried weight composition (%)	Stems	Roots	Leaves
1	Nitrogen	0.2	0.1	0.3
2	Ash	21.20	50.11	13.10
3	Lignin	18.36	15.67	5.49
4	Calorific value (kJ/g-DW)	14.55	9.25	15.33
5	Hemicelluloses	28.35	16.23	31.81
6	Lipid	0.98	0.65	1.93
7	Carbon	29.55	27.22	30.33
8	Protein	7.70	3.33	21.97
9	Cellulose	29.33	18.11	29.86

**Table 2:** Isolated primary metabolite contents (mg/gdw) from different plant parts of *Eichhornia crassipes*.

S.no.	Metabolites (%)	Petiole	Roots	Leaves
1	Carbohydrate	56.33 ± 0.094	38.16 ± 0.102	57.26 ± 0.065
2	Lipid/fat	2.35 ± 0.187	2.79 ± 0.131	5.22 ± 0.110
3	Protein	5.53 ± 0.214	8.48 ± 0.200	15.08 ± 0.084
4	Amino acid	1.53 ± 0.082	1.14 ± 0.126	1.67 ± 0.122
